# Transgender-Specific Differentiated HIV Service Delivery Models in the South African Public Primary Health Care System (Jabula Uzibone): Protocol for an Implementation Study

**DOI:** 10.2196/64373

**Published:** 2024-09-13

**Authors:** Tonia Poteat, Rutendo Bothma, Innocent Maposa, Cheryl Hendrickson, Gesine Meyer-Rath, Naomi Hill, Audrey Pettifor, John Imrie

**Affiliations:** 1 Duke University School of Nursing Durham, NC United States; 2 Wits RHI University of the Witwatersrand Johannesburg South Africa; 3 Division of Epidemiology and Biostatistics, Department of Global Health Faculty of Medicine and Health Sciences Stellenbosch University Stellenbosch South Africa; 4 Health Economics and Epidemiology Research Office Faculty of Health Sciences University of Witwatersrand Johannesburg South Africa; 5 Department of Global Health, Amsterdam Institute for Global Health and Development Amsterdam UMC University of Amsterdam Amsterdam Netherlands; 6 Department of Global Health Boston University Boston, MA United States; 7 South African Centre for Epidemiological Modelling and Analysis Stellenbosch University Stellenbosch South Africa; 8 Department of Epidemiology Gillings School of Global Public Health University of North Carolina at Chapel Hill Chapel Hill, NC United States

**Keywords:** HIV prevention, HIV care, pre-exposure prophylaxis, antiretroviral therapy, gender affirmation, transgender health

## Abstract

**Background:**

Almost 60% of transgender people in South Africa are living with HIV. Ending the HIV epidemic will require that transgender people successfully access HIV prevention and treatment. However, transgender people often avoid health services due to facility-based stigma and lack of availability of gender-affirming care. Transgender-specific differentiated service delivery (TG-DSD) may improve engagement and facilitate progress toward HIV elimination. Wits RHI, a renowned South African research institute, established 4 TG-DSD demonstration sites in 2019, with funding from the US Agency for International Development. These sites offer unique opportunities to evaluate the implementation of TG-DSD and test their effectiveness.

**Objective:**

The Jabula Uzibone study seeks to assess the implementation, effectiveness, and cost of TG-DSD for viral suppression and prevention-effective adherence.

**Methods:**

The Jabula Uzibone study collects baseline and 12-month observation checklists at 8 sites and 6 (12.5%) key informant interviews per site at 4 TG-DSD and 4 standard sites (n=48). We seek to enroll ≥600 transgender clients, 50% at TG-DSD and 50% at standard sites: 67% clients with HIV and 33% clients without HIV per site type. Participants complete interviewer-administered surveys quarterly, and blood is drawn at baseline and 12 months for HIV RNA levels among participants with HIV and tenofovir levels among participants on pre-exposure prophylaxis. A subset of 30 participants per site type will complete in-depth interviews at baseline and 12 months: 15 participants will be living with HIV and 15 participants will be HIV negative. Qualitative analyses will explore aspects of implementation; regression models will compare viral suppression and prevention-effective adherence by site type. Structural equation modeling will test for mediation by stigma and gender affirmation. Microcosting approaches will estimate the cost per service user served and per service user successfully treated at TG-DSD sites relative to standard sites, as well as the budget needed for a broader implementation of TG-DSD.

**Results:**

Funded by the US National Institutes of Mental Health in April 2022, the study was approved by the Human Research Ethics Committee at University of Witwatersrand in June 2022 and the Duke University Health System Institutional Review Board in June 2023. Enrollment began in January 2024. As of July 31, 2024, a total of 593 transgender participants have been enrolled: 348 are living with HIV and 245 are HIV negative. We anticipate baseline enrollment will be complete by August 31, 2024, and the final study visit will take place no later than August 2025.

**Conclusions:**

Jabula Uzibone will provide data to inform HIV policies and practices in South Africa and generate the first evidence for implementation of TG-DSD in sub-Saharan Africa. Study findings may inform the use of TG-DSD strategies to increase care engagement and advance global progress toward HIV elimination goals.

**International Registered Report Identifier (IRRID):**

DERR1-10.2196/64373

## Introduction

### HIV Among Transgender People

Transgender people are a key population with a disproportionate burden of HIV. Globally, transgender women have an estimated HIV prevalence of 19.9%, with 66-fold greater odds of HIV compared with other adults, a prevalence that has not decreased over the past decade despite the introduction of HIV pre-exposure prophylaxis (PrEP) [1,2]. Transgender men also face elevated HIV prevalence, with an estimated global prevalence of 2.6% and 6.8-fold increased odds of HIV [2]. South Africa has the largest HIV epidemic in the world, where more than 7 million people are living with HIV [3]. Approximately 17% of the general population of reproductive age adults have HIV, and prevalence is estimated to be 3-fold higher (58%) among transgender people [4]. A recent integrated biobehavioral respondent-driven sampling study (Botshelo Ba Trans [5]; N=888) estimated HIV prevalence in transgender women to be 46% in Buffalo City and Cape Town and 63% in Johannesburg [6-8]. Unfortunately, transgender men and nonbinary people have been largely overlooked in the HIV response, with no available prevalence data from South Africa.

Engaging transgender people in HIV care and prevention is necessary to reach global goals for ending the HIV epidemic by 2030. Global 2020 targets toward ending the HIV epidemic were missed and often left behind key populations, such as transgender people. For example, in 6 of 13 countries reporting HIV data on transgender people to the Joint United Nations Programme on HIV and AIDS (UNAIDS), less than half of transgender women were able to access HIV prevention services [9]. The Global AIDS Strategy 2021-2026 aims to leave no one behind in the HIV response [10]. Therefore, UNAIDS has set more ambitious targets for 95% of people with HIV to be diagnosed, 95% of diagnosed people with HIV to be on antiretroviral therapy (ART), and 95% of people with HIV on ART to achieve sustained viral suppression (ie, 95-95-95) by 2025 [11]. As key populations accounted for 65% of new HIV infections globally and 35% within sub-Saharan Africa in 2020, UNAIDS emphasized the importance of reaching key populations, including transgender people, to achieve these goals [12,13]. Because stigma and discrimination are known drivers of HIV, UNAIDS also set targets for 2025 that include <10% of health workers reporting negative attitudes toward key populations and <10% of key populations reporting experiences of stigma and discrimination [14]. However, interviews with >1400 transgender people in South Africa in 2023 revealed that only 7% felt safe and comfortable when accessing health care; 72% no longer get services because of disrespect from health facility staff; 65% reported that health facility staff were unfriendly; and 10% had been denied health care outright [15].

Although South Africa has the world’s largest HIV epidemic, it has achieved 95% of people with HIV being aware of their status, 77% of people with HIV on ART, and 71% of people with HIV virally suppressed [3]. Unfortunately, South Africa does not disaggregate HIV continuum data for transgender people. In 2019, a study that combined data from transgender women and men who have sex with men in sub-Saharan Africa (including Cape Town and Soweto, South Africa) found that 56% of people with HIV knew their status, 34% were on ART, and 28% were virally suppressed [16]. Another combined study of transgender women and men who have sex with men in Johannesburg found that 47% were virally suppressed. Modeling data from 2021 indicate South Africa will not reach the HIV elimination threshold (incidence <0.1%) without engaging key populations [17]. Modeling has indicated that halving the rate of ART interruptions in South Africa would substantially reduce new infections, accelerating progress toward ending the HIV epidemic [18].

Available data among transgender men [19] and transgender women [20,21] have found suboptimal levels of ART adherence and viral suppression, even in high-income countries. For example, a US-based study of transgender men with HIV found that while 93% had been prescribed ART, only 60% maintained viral suppression over 12 months [19]. Similarly, a US study of transgender women found that while 80% reported ART adherence, only 59% achieved viral suppression [21]. Disaggregated data on viral suppression among transgender people in South Africa are scant. However, an observational cohort study, including 24 transgender women from South Africa, Kenya, and Malawi found that 25% of transgender women were virally suppressed at baseline and 62.5% were suppressed at 12 months [22].

UNAIDS 2025 targets call for 95% of people at risk for HIV acquisition to use combination HIV prevention, including PrEP [11]. However, PrEP uptake is low among transgender women. A large (N=728) US-based longitudinal study of transgender women’s adherence to PrEP during periods of sexual risk (also known as prevention-effective adherence) found it to be <20% [23]. A smaller South African study (N=213) found that less than half of transgender women who were HIV negative reported they had heard of PrEP and only 11% reported taking PrEP [24]. Qualitative interviews with transgender women in the study (n=36) identified health facility-based antitransgender stigma as a barrier to care seeking [24]. While PrEP data on transgender men in sub-Saharan Africa are unavailable, research in high-income countries has found that PrEP-eligible transgender men report low PrEP use (approximately 11%) [25,26], and health care provider stigma is associated with decreased access to HIV prevention services [27]. Our team found no published data on PrEP engagement among gender nonbinary people.

Multiple studies with transgender people have concluded that stigma and limited access to gender affirmation are associated with increased HIV risk and low engagement in HIV care and prevention services [19,20,28,29]. Access to gender-affirming hormone therapy (GAHT) is a priority for many transgender people, often deemed more important than HIV care or prevention [30]. However, a recent study with transgender women in South Africa found that only 17% had access to GAHT [24]. Research has also found that the use of gender-affirming language by health care providers positively impacts transgender patients’ health care experiences, quality of care, mental health, and likelihood of seeking preventive services [31].

### Differentiated Service Delivery

Transgender-specific differentiated service delivery (TG-DSD) is a promising strategy to overcome barriers to HIV service engagement among transgender people. Differentiated service delivery (DSD) is a term used to refer to client-centered approaches that tailor HIV services to reflect the needs of specific populations [32]. Building blocks of DSD include adaptations to when (eg, frequency of clinical visits), where (eg, health facility or community), by whom (eg, nurses or community health workers), and what (eg, ART, PrEP, or counseling) services are offered based on the needs of the individual client [33]. When implemented at scale, DSD is intended to simplify and adapt HIV services across the prevention and treatment continuum in ways that better serve client needs, improve client outcomes, and reduce burdens on the health system [32]. UNAIDS 2025 targets call for 90% of people with HIV and people who face heightened vulnerability to HIV acquisition to be linked to people-centered, context-specific, integrated services, that is, DSD [11].

DSD sites may differentiate services according to client clinical characteristics (eg, newly diagnosed with HIV or stable on ART), subpopulations to which they belong (eg, pediatric, pregnant, or key population), and context in which they live (eg, concentrated versus generalized epidemic and rural versus urban setting) [33]. The World Health Organization (WHO) promotes the use of DSD for key populations to increase service acceptability, quality, and coverage, and reduce costs [34]. Facilities across sub-Saharan Africa have implemented a variety of DSD models for different subpopulations [35-37]. However, South Africa is the first in the region to implement TG-DSD models that tailor services to the specific needs of transgender people, including the need for GAHT.

As GAHT is a priority for transgender people, guidance on DSD for key populations from the WHO [38] and the International AIDS Society [33] recommend the inclusion of GAHT in DSD for transgender people. Promising research in Peru, Thailand, and the United States suggests that integration of GAHT services with HIV care and prevention can improve engagement with HIV services [39-42]. However, the evidence base is limited to case studies or protocols lacking outcome data [39,41,43,44], cross-sectional associations between the use of GAHT and engagement in HIV services [45,46], or outcomes and perceptions of services at a single site [40,42,47]. No published studies compare HIV outcomes at TG-DSD sites versus standard service delivery (SSD) sites, and no published studies of TG-DSD have been conducted in sub-Saharan Africa, the region with the greatest burden of HIV [35].

A recent review of DSD models called for a deeper understanding of their mechanism of effect and noted that while the success of DSD implementation may differ based on a variety of factors, few studies have evaluated comparative implementation or cost [48]. To inform ongoing efforts to achieve UNAIDS goals and leave no one behind, data are needed on the effectiveness of TG-DSD models for improving engagement in HIV services and on implementation barriers, facilitators, acceptability, feasibility, and cost.

Wits RHI demonstration sites provide a unique opportunity to evaluate TG-DSD in sub-Saharan Africa [49]. Over the last several years, Wits RHI has collaborated with transgender-led organizations in 4 municipal areas (Gauteng, Cape Town, Nelson Mandela Bay, and Buffalo City) in South Africa to implement a TG-DSD site in each area (n=4 sites). Each site integrates gender-affirming care, including GAHT, with a full package of facility-based and community-based (mobile) HIV care and prevention services, consistent with the WHO key population and DSD guidelines [49-51]. All staff members receive training on the provision of nonstigmatizing, gender-affirming services. Clinicians receive additional competency training in gender-affirming medical care. These real-world sites, currently supported by programmatic (nonresearch) funds from United States Agency for International Development, provide an important opportunity to conduct much-needed implementation research [32,34,52]. Findings will not only directly inform the future direction of DSD strategies in South Africa but also advance the knowledge base on *if* and *how* TG-DSD models work and the cost of implementation.

A robust evidence base supports DSD among nonkey populations. A systematic scoping review of DSD for HIV treatment included 40 publications (18 from South Africa) involving over 240,000 participants spanning 9 countries in sub-Saharan Africa [35]. The review found that a variety of DSD models were effective for improving viral suppression and retention in care compared with SSD. DSD improved acceptability and, in some cases, reduced the cost of care to clients. While these data provide a strong scientific premise for evaluating DSD models, none of the evaluated DSD models focused on key populations.

### Study Aims and Hypotheses

This study, titled Jabula Uzibone (a Zulu phrase that can be translated into English as “be happy, see yourself”), is designed to fill an important gap in data on implementation, effectiveness, and cost of TG-DSD models in sub-Saharan Africa. It has the aims presented in Textbox 1.

**Jabula Uzibone study aims.**

**Aims**

- Aim 1: assess the barriers, facilitators, acceptability, and feasibility of transgender-specific differentiated service delivery (TG-DSD).
- Aim 2: evaluate the effect of TG-DSD on viral suppression and prevention-effective adherence and test stigma and gender affirmation as mediators.
  - Hypothesis 1: rates of viral suppression (HIV RNA <50 copies/mL) are more likely to increase among transgender people on antiretroviral therapy (ART) at TG-DSD sites than among transgender people on ART at standard service delivery (SSD) sites over the 12-month study period.
  - Hypothesis 2: rates of prevention-effective adherence (measured by tenofovir levels >700 fmol/punch among participants on pre-exposure prophylaxis [PrEP] or no condomless intercourse among participants who are HIV negative and not taking PrEP) are more likely to increase among participants at TG-DSD sites than among those at SSD sites over the 12-month study period.
  - Hypothesis 3: stigma and gender affirmation will mediate relationships between service delivery models (TG-DSD vs SSD) and HIV outcomes (viral suppression and prevention-effective adherence).
- Aim 3: estimate the cost associated with TG-DSD versus SSD using a microcosting approach to determine the cost per service user served and per service user successfully treated at TG-DSD sites relative to SSD sites, as well as the budget needed for successful South Africa–wide implementation.

## Methods

### Overall Study Design

Jabula Uzibone is an observational, multisite, mixed methods, and prospective implementation study. To meet the first aim, we will assess the implementation of TG-DSD models and SSD models at the facility level using standardized observation checklists at 8 facilities (1 DSD site and 1 SSD site per area in all 4 areas). We will examine implementation, acceptability, feasibility, and barriers to and facilitators of TG-DSD via key informant interviews (KIIs; n=6 per site) with health facility staff. At the client level, we will conduct in-depth interviews (IDIs) with 30 transgender people (15, 50% from DSD sites and 15, 50% from SSD sites) drawn from the longitudinal cohort enrolled for aim 2 described in the next paragraph.

To meet the second aim, we will enroll a cohort of ≥600 transgender people and follow them for 12 months. Of the transgender people enrolled, ≥400 (66.7%) will be transgender people living with HIV. This will include ≥200 (50%) who receive ART at TG-DSD sites and ≥200 (50%) who receive ART at SSD sites in the same area as the TG-DSD sites. We will also enroll ≥200 transgender people without HIV. This will include ≥100 (50%) participants without HIV at TG-DSD sites and ≥100 (50%) without HIV at SSD sites in the same area as the TG-DSD sites. Each participant will complete a quarterly survey to assess engagement in HIV prevention and care, changes over time in experiences of gender affirmation, and experiences of stigma.

For participants on ART, we will measure HIV RNA levels (ie, viral load) at baseline and 12 months. For participants on PrEP, we will measure tenofovir levels at baseline and 12 months. For participants who are HIV negative and not on PrEP, we will assess self-reported condomless sex. Prevention-effective adherence is defined as being HIV negative and meeting one of the following criteria: (1) adherent to PrEP based on tenofovir levels, (2) self-report of consistent condom use in the prior 30 days, or (3) self-report of no anal or vaginal sex in the prior 30 days. Table 1 presents a list of data collection activities organized by level of analysis, participant type, and month of enrollment.

**Table 1:** Data collection activities by level of analysis, participant type, and month of enrollment.

Level			Participant, n (%)		Baseline		Months 3, 6, and 9		Month 12
**Facility level (n=8)**									
	TG-DSD^a^ sites	4 (50)		Checklists		Quantitative survey		KIIs^b^ (6/site, n=24)	
	SSD^c^ sites	4 (50)		Checklists		Quantitative survey		KIIs (6/site, n=24)	
**Transgender people** **living with HIV (n=400)**									
	Care at TG-DSD sites	200 (50)		HIV RNA level		Quantitative survey		IDI^d^ (n=15)	
	Care at SSD sites	200 (50)		HIV RNA level		Quantitative survey		IDI (n=15)	
**Transgender people** **without HIV (n=200)**									
	Care at TG-DSD sites	100 (50)		Tenofovir-level		Quantitative survey		IDI (n=15)	
	Care at SSD sites	100 (50)		Tenofovir-level		Quantitative survey		IDI (n=15)	

^a^TG-DSD: transgender-specific differentiated service delivery.

^b^KII: key informant interview.

^c^SSD: standard service delivery.

^d^IDI: in-depth interview.

To meet the third aim, we will estimate the incremental costs of DSD implementation compared with SSD from the provider perspective by conducting a bottom-up analysis of the resources used and costs accrued by the TG-DSD cohort and comparing them to the costs of SSD established in recent studies [53,54]. We will also calculate the cost per successful outcome (suppressed viral load for those on ART and prevention-effective adherence for those without HIV) and the total budget required for a national roll out of TG-DSD.

### Ethical Considerations

Ethics approval was received for this study from the University of Witwatersrand Human Research Ethics Committee (M220420) in June 2022 and from the Duke University Health System institutional review board (Pro00113320) in June 2023. All participants complete a written informed consent process, including the option to opt-out of participation, after eligibility screening and before any data collection activities. All data are collected in a private room at the study site or a private area (eg, mobile van) in the community. Name and contact information are collected in order to send reminders of follow-up study visits. However, personal identifiers are retained separate from study data. Each participant is assigned a unique anonymous study identifier that is associated with their data. Personal identifiers are only accessible by specific study staff who have permission to contact participants for reminder purposes. All study staff have been trained in human subjects protections and good clinical practices. Participants receive ZAR 300 (US $17) at the end of each study visit as well as light refreshments (eg, a sandwich).

### Study Frameworks

This study uses the Reach, Effectiveness, Adoption, Implementation, and Maintenance (RE-AIM) [55,56] implementation science evaluation framework and the gender affirmation framework [57]. Data collection and analysis for all aims are guided by RE-AIM. Key measures and analyses for aim 2 are also guided by the gender affirmation framework, a conceptual framework that outlines mechanisms by which TG-DSD is expected to improve HIV outcomes.

RE-AIM is often used to guide the evaluation of implementation strategies [55]. It is well-suited for studies of projects that are in the implementation process [56] and guides our selection of study measures [58]. The first 4 RE-AIM domains are listed in Textbox 2, with examples of research questions for each domain. These domains (reach, effectiveness, adoption, and implementation) are most applicable to this study, while the fifth domain, maintenance, will not be addressed.

**Reach, Effectiveness, Adoption, Implementation, and Maintenance (RE-AIM) domains applied in this study and example research questions.**

**Domains and example research questions**

- Reach: Is the intervention reaching the desired population?
- Effectiveness: Does the intervention accomplish its goals?
- Adoption: To what extent are the people who should deliver the intervention participating?
- Implementation: To what extent is the intervention consistently implemented?

The gender affirmation framework provides a model for drivers of HIV outcomes. Gender affirmation is a process of recognition and support for one’s gender identity [57]. Gender affirmation has 4 core facets: medical (eg, GAHT and surgery), psychological (eg, self-acceptance and sense of alignment with one’s gender), social (eg, use of desired name and pronouns and wearing clothes associated with one’s gender identity), and legal (eg, change of name and gender marker on legal documents) [59].

The gender affirmation framework posits that antitransgender stigma leads to social oppression and psychological distress. Social oppression reduces access to gender affirmation while psychological distress increases the need for gender affirmation. The gap between access and need drives behaviors that increase HIV vulnerability. Significant relationships between unmet gender affirmation needs and HIV risk behavior as well as ART interruptions are well documented [57,60-63]. This framework informs our hypothesis that TG-DSD will reduce stigma and increase gender affirmation, thereby increasing engagement in HIV services and improving HIV outcomes (ie, viral suppression and prevention-effective adherence) among transgender people. Our measures of stigma as well as medical (ie, GAHT) and psychological gender affirmation will be used to test hypothesis 3 for aim 2.

### Transgender-Specific DSD Models

Wits RHI provides TG-DSD models at 4 sites. Of these, 2 (50%) are stand-alone sites and 2 (50%) are in park homes on an SSD facility campus. All TG-DSD sites offer GAHT in an integrated HIV and primary care package, which includes both facility-based (fixed) services and community-based (mobile) services. All services are free to clients.

Details of the care provided at those sites have been published elsewhere [49], and an overview is provided in Table 2 and Textbox 3. Each site employs peer navigators, community liaison officers, professional nurses, a social worker, a site manager, and community health workers. Each site also employs a sessional physician and psychologist. Services have been designed and delivered in collaboration with local transgender community–based organizations. All cadres of staff (clinical, nonclinical, professional, and lay) complete transgender sensitization training conducted by transgender-led community organizations. These trainings build staff skills in providing nonstigmatizing, transgender-friendly services. Trainings take place upon hire and routinely thereafter during in-service sessions.

**Table 2:** Wits RHI transgender-specific differentiated service delivery and models of care in South Africa.

	Guateng	Cape Town	Nelson Mandela Bay	Buffalo City
Model	Stand-alone	Stand-alone	Colocated with SSD^a^	Colocated with SSD
Population density	Urban	Urban	Peri-urban and rural	Peri-urban and rural
GAHT^b^	Provided on site	Provided on site	Provided on site	Provided off site

^a^SSD: standard service delivery.

^b^GAHT: gender-affirming hormone therapy.

**Components of Wits RHI transgender-specific differentiated service and their descriptions.**

**Service and description**

- Clinical services: primary health care, sexually transmitted infection screening and treatment, tuberculosis screening, HIV treatment and prevention, and gender-affirming hormone therapy (GAHT)
- Ancillary services: psychosocial support, referral to postviolence care, legal support, and substance use services
- Full-time staff: 4 to 10 peer navigators, 0 to 2 community liaison officers, 1 social worker, 0 to 2 community health workers, 2 nurses, and 1 site manager
- Sessional staff: 1 physician and 1 psychologist
- Community partnerships: local transgender community organizations and local AIDS councils
- Community leadership: community advisory boards comprising transgender advocates and activists who guide and monitor services

Where possible, transgender people are appointed in all staff cadres. Most community health workers and peer navigators are transgender people. Program-specific Facebook (Meta Platforms, Inc) and WhatsApp (Meta Platforms, Inc) groups provide information on services and events to the community. Transgender-friendly health information, education, and communications printed materials are available.

Clinical services related to GAHT are provided only at the facility. The client comes to the facility monthly for the first 3 months on hormones for laboratory testing, side effect assessment, and dose adjustments. After the third month, when clients are on a stable dose, they return to see the medical provider at month 6, then every 6 months subsequently for monitoring. Multi-month dispensing of hormones is currently a challenge in South Africa due to stock shortages; therefore, clients on GAHT return monthly for dispensing visits only.

In addition to facility-based GAHT and HIV services, each TG-DSD site provides mobile HIV services in the community throughout their catchment area. These mobile services are offered in a van equipped with examination rooms and basic medical equipment. Mobile teams include a peer navigator, a community health worker, and a professional nurse. They offer primary health services, HIV testing and counseling, and multi-month dispensing of ART and PrEP.

### SSD Models

Public primary health care centers in South Africa provide SSD through a professional nurse-based, physician-supported infrastructure of >3500 clinics and community health centers, available within 5 km of more than 90% of the population and free at the point of care [64]. Primary health care centers are the first point of entry for health care access. These facilities provide a comprehensive package of basic services, including maternal, child, and reproductive health, and HIV and tuberculosis testing and treatment, including ART, screening and care for noncommunicable diseases, and treatment of common ailments. The primary health care system is supported by community teams, referred to as ward-based primary health care outreach teams. These teams are allocated to specific wards according to the size, geography, and social and structural considerations. They provide health information to the community, and screen and refer those who need further management to corresponding primary health care facilities [65]. Despite a progressive constitutional and legal framework, transgender people continue to face stigma and discrimination when they access SSD sites for health care [66-68]. GAHT is not available in public primary health care facilities. In general, access to GAHT is limited in South Africa, where it is only provided by specialist endocrinologists at tertiary facilities; and transgender people may face waiting times of up to 20 years or more to access gender-affirming surgery in the public sector [69,70].

### Study Procedures

#### Facility-Level Eligibility, Recruitment, and Data Collection

We have enrolled all 4 Wits RHI TG-DSD sites listed in Table 2. In collaboration with district health departments, we have recruited 1 SSD site within the catchment area of each DSD site for a total of 4 TG-DSD sites and 4 SSD sites. SSD sites are similar to TG-DSD sites in urbanicity, socioeconomic status of the client population, and types of HIV services offered (ie, both ART and PrEP). We shared information about the study with the district and SSD site leadership and invited them to participate in the study. Study staff will visit all 8 recruited sites (TG-DSD and SSD) to complete a standardized observation checklist at baseline and 12 months. Checklist items include the number, type, and role of staff; content and frequency of staff training; facility hours, amenities (eg, number of rooms), and community outreach efforts; standard policies and procedures for delivery of HIV and sexually transmitted infection testing, PrEP, ART, and GAHT services, including, but not limited to counseling, prescribing, dispensing, laboratory monitoring, tracking, and visit frequency; sex and gender documentation on clinical forms and records; the number of transgender clients; number of transgender people prescribed GAHT; number of transgender people prescribed PrEP; number of transgender people prescribed ART; availability and use of psychosocial services; and cost of service delivery.

Leadership at each recruited site facilitate recruitment of 6 staff members per site (n=48) to complete a KII at baseline and 12 months. Eligible staff members are aged ≥18 years and work for 1 of the TG-DSD sites or a selected SSD site. We enroll 1 person in each role at each site, that is, peer navigator, community liaison officer, social worker, community health worker, nurse, and site manager. If a staff member leaves between baseline and 12 months, we will interview another person in the same role for the 12-month time point. We contact potential key informants via email or WhatsApp and invite them to participate.

An experienced qualitative researcher conducts the KIIs after the informed consent process. A semistructured KII guide will be used to organize the interview. KIIs last approximately 60 minutes and include open-ended questions that explore facilitators, barriers, strengths, and weaknesses of the DSD and SSD approaches and recommendations for improving service delivery. At the end of the qualitative interview, participants complete a brief, interviewer-administered quantitative tool measuring antitransgender stigma [71] and validated measures of intervention acceptability, feasibility, and appropriateness [72,73]. KIIs are audio-recorded with permission of the participant, and notes are taken during and after the interview to facilitate accuracy and appropriate analysis. Where possible, interviewers are fluent in local languages. When interpretation is required, a trained study staff member interprets during the interview.

We collect data on the costs of implementation at all TG-DSD sites by conducting a bottom-up analysis of the resources used and costs accrued by all transgender cohort participants who receive TG-DSD. We also collect facility-level data on overhead cost items, such as space, administration, and utility costs, and allocate these to the service and our cohort based on the number of visits. We use our extensive database of public sector prices and salaries, as well as information on staff levels at each site, to estimate the public sector costs; these are data we have used in other costing studies in South Africa [53,74,75].

In collaboration with local transgender organizations, trained transgender people recruit their peers from their social networks via social media posts, WhatsApp groups, and word-of-mouth. Eligibility criteria include age ≥18 years, identity as a gender different from the sex assigned at birth, and residence in the catchment area for 1 of the enrolled health facilities. Participants must either be living with HIV and prescribed ART or HIV negative and PrEP eligible (whether or not they are currently taking PrEP). We use stratified sampling to ensure enrollment of ≥400 participants on ART and ≥200 participants who are HIV negative. Within those strata, we further ensure that half are individuals who receive care at a TG-DSD site and the other half are transgender people who receive care at an SSD site in the same catchment area. We use quotas to ensure that no TG-DSD site makes up <20% of TG-DSD participants. At the enrollment visit, participants are rescreened for eligibility, including rapid HIV testing to confirm self-reported HIV status.

#### Client-Level Eligibility, Recruitment, and Data Collection

Participants complete study data collection visits with the study team in a dedicated room at the site or in the community. Over the 12-month study period, participants complete 5 study visits: baseline, 3, 6, 9, and 12 months. At each visit, trained study staff use electronic tablets to administer quantitative surveys lasting 30 minutes. Surveys assess the key measures listed in Table 3. Survey data are collected and managed using REDCap (Research Electronic Data Capture; Vanderbilt University), which synchronizes with the encrypted cloud-based server at Wits RHI, where it is stored for analysis.

**Table 3:** Key quantitative survey measures.

Construct		Measure	Citation
**Implementation outcomes**			
	Acceptability	Acceptability of Intervention Measure	Weiner et al [73], 2017
	Feasibility	Feasibility of Intervention Measure	Weiner et al [73], 2017
	Appropriateness	Intervention Appropriateness Measure	Weiner et al [73], 2017
**Enacted stigma**			
	Health facility stigma	Brief Health Facility Stigma Tool	Nyblade et al [71], 2013
**Experienced stigma**			
	Transgender-specific stigma	Gender Minority Stress and Resilience measure	Testa et al [76], 2015
	Intersectional stigma	Intersectional Discrimination Index	Scheim and Bauer [77], 2019
**Gender affirmation**			
	Psychological	Psychological Gender Affirmation Scale	Sevelius et al [78], 2021
**Prevention-effective adherence [23]**			
	Sexual behavior	“Have you had anal or vaginal sex in the past 30 days?”	—^a^
	Condom use^b^	“How often did you use condoms when you had sex?”	—
	PrEP^c^ adherence^d^	“How many days have you missed PrEP pills in the last 30 days?”	—

^a^No relevant citation.

^b^This question is only asked of participants who report sex in the past 30 days.

^c^PrEP: pre-exposure prophylaxis.

^d^This question is only asked of participants who self-report taking pre-exposure prophylaxis.

At the baseline and 12-month visits, all participants have blood drawn by a professional nurse for laboratory analysis. Serums from participants on ART are tested for HIV RNA. HIV RNA levels <50 copies/mL are the threshold for viral suppression, consistent with South African national guidelines [79]. Dried blood spots from participants on PrEP will be tested for tenofovir diphosphate levels [80,81]. Levels >700 fmol/punch are consistent with ≥4 doses per week and will be used as a marker for adherence [82,83].

Thirty (5%) of the 600 enrolled transgender participants who complete the quantitative survey and laboratory analyses at baseline are invited to participate in longitudinal qualitative IDIs at baseline and 12 months. IDIs are most appropriate for eliciting detailed accounts of participant experiences and perceptions of service delivery models. The open-ended nature of IDIs provide the opportunity to more deeply explore issues relevant to the study aims. Embedding qualitative data collection within implementation research can enrich the understanding of how and why service delivery models work or do not work [84,85]. Longitudinal interviews are important to study how participants experience, interpret, and respond to the service delivery model over time [86].

We invite participants to take part in IDIs using stratified purposive sampling [87]. We stratify by care model, that is, 15 who receive care at TG-DSD sites and 15 who receive care from SSD sites. Within each stratum, we purposively sample to ensure diversity by HIV status, catchment area, and gender identity (transgender women, transgender man, and gender nonbinary). A topical guide structures the interview. Open-ended questions, followed by prompts, are used to elicit participant narratives. Study staff with training and experience in qualitative research conduct the IDIs. Each IDI explores perceptions of DSD versus SSD models of care, satisfaction or concerns related to type of care received, experiences with GAHT, if any, experiences with ART or PrEP, as appropriate, and recommendations for service improvement. Follow-up IDIs will explore how participant experiences and perceptions have changed over time and provide an opportunity to clarify earlier responses. With participant permission, all interviews are digitally audio-recorded. Interviewers write field notes and narrative summaries after each interview that supplement the transcripts. Participants’ qualitative data are linked with the survey data to allow for integration with their quantitative measures of stigma and gender affirmation.

Participant tracking and retention rely on multiple strategies. At enrollment and each subsequent study visit, we document contact details for participants (eg, phone numbers, addresses, WhatsApp, and social media profiles) to communicate study reminders and re-engage participants who may miss a study visit. If participants are unable to come to the study site for data collection, research staff support retention by accompanying mobile health facility staff into the community and collecting study data there. We provide modest financial remuneration and refreshments to incentivize participation and retention. We have hired transgender study staff to create a welcoming and affirming environment for study participation. Gender-affirming stigma reduction training of research staff facilitates strong staff-participant rapport to encourage retention. Participants are provided a referral to counseling services if they demonstrate psychological distress while engaging with study staff.

### Overall Approach to Data Analysis

Data analysis will include both qualitative and quantitative data. Table 4 outlines the relationships between the RE-AIM framework domains and the types of data to be collected.

**Table 4:** Relationship among the Reach, Effectiveness, Adoption, Implementation, and Maintenance domains; study aims; and data collection.

Domains (aim), levels, data type, and source				Example qualitative questions and quantitative measures
**Reach (1)**				
	**Facility**			
		Site checklist	Number of transgender people who receive GAHT^a^, ART^b^, or PrEP^c^ from the site	
		KIIs^d^ with health staff	What factors contribute to the engagement of transgender people at TG-DSD?	
**Adoption (1)**				
	**Facility**			
		KIIs with health staff	What barriers and facilitators affected staff implementation of TG-DSD?	
**Implementation (1 and 3)**				
	**Facility**			
		Site checklist	Number and type of staff and their roles in implementation. Availability of GAHT on site. Frequency of stigma reduction training. Cost of service delivery (infrastructure, labs, visits, etc)	
		KIIs with health staff	How was TG-DSD implemented and adapted over time? AIM^e^, IAM^f^, and FIM^g^ implementation science measures	
	**Client**			
		IDIs^h^ with transgender participants	Tell me about the services you received at the site?	
**Effectiveness (2)**				
	**Facility**			
		KIIs with health staff	What changes in use of HIV services (eg, ART and PrEP uptake and adherence) did you notice when TG-DSD was implemented? Health Facility Stigma Scale by Nyblade [71]	
	**Client**			
		IDIs with transgender participants	Tell me about your experience receiving care at the site.	
		Survey with transgender participants	Intersectional stigma scale by Scheim and Bauer [77], Psychological Gender Affirmation scale by Sevelius et al [78], and GAHT use by self-report.	
		Laboratory test with transgender participants	HIV RNA level by serum analysis (for transgender participants on ART) and tenofovir level by DBS^i^ (for transgender participants on PrEP)	

^a^GAHT: gender-affirming hormone therapy.

^b^ART: antiretroviral therapy.

^c^PrEP: pre-exposure prophylaxis.

^d^KII: key informant interview.

^e^AIM: Acceptability of Intervention Measure.

^f^IAM: Intervention Appropriateness Measure.

^g^FIM: feasibility of intervention measure.

^h^IDI: in-depth interview.

^i^DBS: dried blood spot.

Qualitative interview (KII and IDI) recordings will be transcribed and translated into English. The transcripts, field notes, and narrative summaries will be uploaded into qualitative data management software, Atlas.ti (ATLAS.ti Scientific Software Development GmbH), to facilitate analyses. During regularly scheduled meetings, the study team will review field notes, transcripts, and summaries; discuss emerging themes; and revise interviewing and coding strategies, as needed.

Statistical analysis of quantitative data will be performed using Stata (StataCorp) and MPlus (Muthén & Muthén). Data exploration of key variables will be conducted to assess for implausible values and determine whether statistical assumptions (eg, normal distributions of data) for planned statistical analyses are satisfied. Categorical variables will be reported as frequencies and percentages, with means and SDs or medians and IQRs reported for continuous variables. Patterns of missing data will be examined, including testing for differences between participants with and without missing data. A series of sensitivity analyses will be conducted to evaluate the robustness of conclusions drawn from the primary models to departures from the missing at random assumption by comparing the magnitude of the primary effect. In the rare situation that >10% missing data is observed, we will use multiple imputation, as appropriate. We will specify 2-sided tests, .05 significance level (α=.05), and compute 95% CIs throughout.

### Qualitative Data Analysis for Aim 1

Qualitative transcripts from the KIIs with health facility staff and IDIs with transgender people will be analyzed to assess barriers, facilitators, acceptability, and feasibility of TG-DSD. To identify preliminary results quickly, we will use a framework-guided rapid analysis in which transcripts are summarized in a structured template [88,89]. The template will include a section for each of the 4 aspects of this aim: barriers, facilitators, acceptability, and feasibility.

For full analysis, a priori codes based on the interview guides and key implementation constructs from RE-AIM will be used to generate the initial codebook [84]. A total of 2 independent analysts will code a subset of transcripts and meet weekly to discuss the textual context, update the codebook, assess interrater reliability, and generate analytic memos. Once the codebook is finalized, the remainder of the transcripts will be systematically coded. Code summaries will be consolidated into matrices by each category of variation (eg, site) to identify salient themes [90]. Any differences in code density will be explored after coding completion from baseline to 12 months and by site characteristics, such as district, urbanicity, and population size.

Codes and associated quotes will be examined within and across transcripts to identify patterns and recurrent themes related to the implementation constructs [91]. After preliminary findings have been shared with key stakeholders for member-checking [92], deductive in-depth content analyses will be conducted with open coding followed by focused coding. Once coding is complete, we will use visualization features of Atlas.ti to map out differences in code density by service delivery model.

To take advantage of the longitudinal nature of the data, we will apply multiple analytic approaches to the constant comparison technique as outlined by Lewis [93]. We will review the narrative summaries for each participant over time to look for within-individual changes. We will read across the transcripts for each period to look for themes unique to specific periods, and we will compare all data across service delivery models (TG-DSD vs SSD). Analytical memos will describe emergent themes and track the analytic process. Peer debriefing, triangulation with quantitative data, member-checking, and memos will support rigor [94].

Sample sizes for qualitative inquiries are designed to collect enough data to reach theoretical saturation, defined as the point at which no new themes emerge from ongoing data collection [95]. Samples sizes as low as 6 can achieve saturation when the study population is homogeneous. However, a sample size of 12 to 20 is typically needed, fewer for longitudinal studies. To account for diversity by site, role, gender identity, education level, etc, we have chosen a sample size of 48 individuals for KII participants, for a total data set of 96 KIIs (including baseline and 12 months). We have selected a smaller sample size of 15 per arm (n=30) for IDIs with transgender clients as they will share similarities which will produce a robust data set of 60 total IDIs for analysis.

### Quantitative Data Analysis for Aim 1

Standardized checklists from DSD and SSD sites will be examined using descriptive statistics. We will perform chi-square tests or Fisher exact tests for associations between categorical variables. We will perform unpaired Student *t* tests to compare continuous variables or outcomes between 2 groups. We will perform Wilcoxon rank sum tests, if data are not normally distributed.

In the DSD sites, to assess acceptability and perceived feasibility of implementing the differentiated care model, staff at each implementing site will complete a brief survey with implementation outcome measures at 12 months. The Acceptability of Intervention Measure (AIM), the Intervention Appropriateness Measure (IAM), and the Feasibility of Intervention Measure (FIM) will be used to quantitatively measure implementation outcomes related to the TG-DSD model [73]. Each measure consists of 4 items, with response options on a Likert scale from 1 (completely disagree) to 5 (completely agree), for a possible summary score range from 4 to 20. Higher scores indicate greater acceptability and feasibility.

Descriptive statistics will be used to characterize each measure overall and by site. On the basis of a score of 4 (agree) for all 4 items in the scale, a summary score ≥16 on each scale would be consistent with overall agreement that the strategy was acceptable or feasible, respectively [73]. Therefore, we will test the hypothesis that the overall mean score for each measure (AIM and FIM) is ≥16 using a 1-sample *t* test. If score data deviate from normality (skewed or nonnormal), we will conduct a 1-sample Wilcoxon signed test. We will conduct a secondary analysis to test for significant changes in baseline and 12-month scores across sites using paired sample *t* tests for normally distributed data or Wilcoxon signed rank tests if the data are nonnormal.

Using data from both study arms, facility-based stigma will be measured using the brief health facility stigma tool [71]. Each item includes 4 response options on a Likert scale that ranges from “never” to “most of the time.” Higher scores indicate greater facility-based stigma. Descriptive statistics will be generated as outlined earlier for the implementation outcome measures. We will compare mean summary scores at TG-DSD sites versus SSD sites at baseline and at 12 months, using 2-sample *t* tests if normally distributed or Wilcoxon rank tests if nonnormal. Exploratory analyses will include Pearson correlation analyses and bivariate regression to assess associations between site characteristics (eg, location and urbanicity), including facility-based stigma scores and the aforementioned implementation outcome scores.

### Qualitative and Quantitative Data Integration for Aim 1

Qualitative and quantitative data will be integrated using results-based convergent synthesis in which they will be analyzed separately then merged using joint display [96-98]. Qualitative themes will triangulate and provide context for facility stigma scores and implementation measures. Data from the checklists will be integrated into the joint display to assess visually any relationships between site features and the relationship between TG-DSD and HIV outcomes. Integrated displays will be used to identify implementation patterns. The qualitative code density (frequency of each code) will be displayed by service delivery model (TG-DSD vs SSD). Visualization will facilitate identification of similarities and differences in participant experiences across models.

### Data Analysis for Aim 2

We will use quantitative methods to evaluate the effectiveness of TG-DSD for viral suppression and prevention-effective adherence and to assess stigma and GAHT use as potential mediators. As noted earlier, our first hypothesis is that rates of viral suppression are more likely to increase among transgender people on ART at TG-DSD sites than among transgender people on ART at SSD sites over the 12-month study period.

The primary outcome measure is the proportion of transgender people on ART with viral suppression (HIV RNA <50 copies/mL). Descriptive analyses will summarize HIV viral suppression comparing participants at TG-DSD versus SSD sites as well as by gender identity at baseline and 12 months. Percent distributions will be computed for categorical variables and means, ranges, SDs, medians, and IQRs will be presented for continuous variables stratified by attendance at TG-DSD versus SSD sites. The primary estimate for this aim is the absolute difference in the change in proportion of virally suppressed during the study period between participants at TG-DSD sites and those at SSD sites. A difference-in-difference (D-i-D) analysis will be conducted to account for anticipated differences between groups at baseline (TG-DSD vs SSD). In addition, we will compare differences at 12 months considering baseline values to enhance the robustness of the estimators of the differences in the 2 groups.

A mixed-effects binomial model will be fit, where the binary viral load suppression status of each client is regressed on fixed effects for time (baseline vs 12 months), TG-DSD versus SSD site, the time by site model interaction, and client-level covariates (age and gender identity). In addition, random intercepts for each of the sites will be included in the model to account for within-site correlation. The D-i-D estimator is the estimated regression coefficient for the time by service delivery model interaction term. All models will account for clustering and be adjusted for relevant confounders in baseline covariates.

The second hypothesis is that rates of prevention-effective adherence are more likely to increase among transgender people who are HIV negative at TG-DSD sites than among those at SSD sites, over the 12-month study period. We will follow the same steps outlined above for the primary hypothesis. However, we define the outcome measure (prevention-effective adherence) as the proportion of (1) participants on PrEP with TFV levels >700 fmol/punch and (2) participants who are not on PrEP who report no condomless intercourse during the prior 30 days. The estimate will be the absolute difference in the change in the proportion who achieve prevention-effective adherence during the study period comparing those at TG-DSD versus SSD sites. In addition, we will estimate the difference between TG-DSD and SSD sites at 12 months, considering baseline values.

Clients who receive SSD services on enrollment may switch to TG-DSD over time, thereby reducing the sample size of the SSD arm and creating a threat to internal validity. To identify and account for switches over time, we will update information about where the participant is receiving care at every study visit. Participants who transfer from an SSD site to a TG-DSD site will be censored after the date of their last visit for the primary analysis. It would be inappropriate and unethical to require participants to continue receiving care at a specific site, therefore we will use careful tracking to inform an exploratory dose-response analysis that considers the amount of time a participant received care at the TG-DSD sites. The amount of time receiving care at the TG-DSD site will be the independent variable, with the change in outcome from baseline to 12 months as the dependent variable.

**Figure 1:**
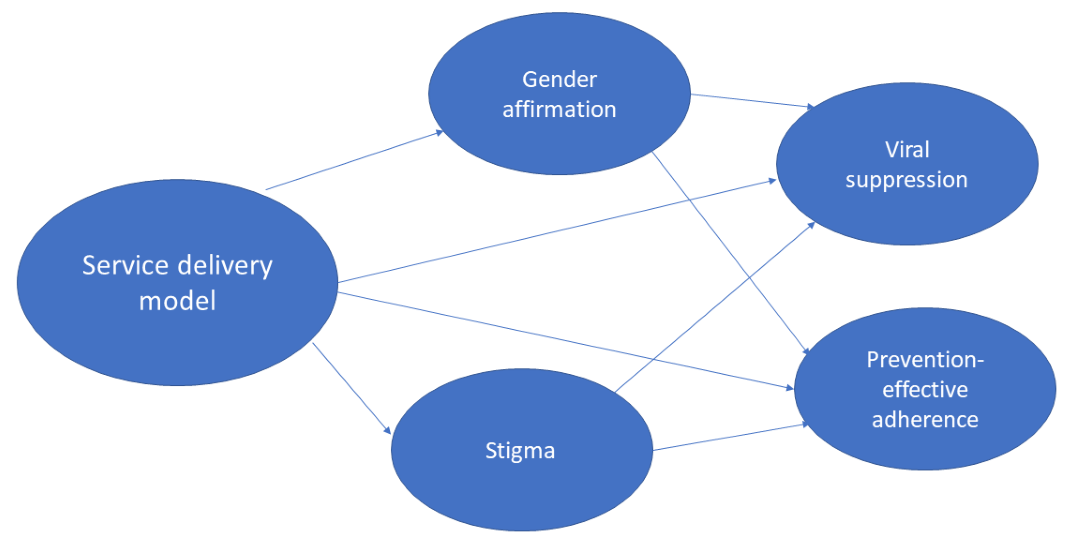
Structural equation model to test stigma and gender affirmation as mediators of relationships between service delivery type and HIV outcomes.

The third hypothesis is that stigma and gender affirmation will mediate relationships between service delivery models (TG-DSD vs SSD) and HIV outcomes (viral suppression and prevention-effective adherence). Mediation analyses will be used to investigate whether any association between service delivery models and HIV outcomes are significantly mediated by stigma (measured using Nyblade et al [71], Testa et al [76], and Scheim and Bauer [77] scales in Table 3) or gender affirmation (measured using the proportion of participants on GAHT and the Sevelius et al [78] gender affirmation scale in Table 4). Structural equation modeling will be used for this analysis. Structural equation modeling provides tests of the direct and indirect paths from the independent variable to the dependent variables [99]. The independent variable will be the service delivery model (TG-DSD vs SSD), and the dependent variables will be change from baseline to 12 months in the mediators (stigma and gender affirmation measures) and change from baseline to 12 months in the outcomes (difference in viral suppression and difference in prevention-effective adherence). Structural equation models will test direct paths (eg, from serviced delivery model to viral suppression) and indirect paths (eg, from service delivery model to stigma and from stigma to viral suppression). The basic model to be tested for this analysis is in Figure 1. We will assess standard fit statistics for the overall model: model chi-square, adjusted goodness of fit, comparative fit index, and standardized root mean square error of approximation.

### Exploratory Analyses for Aims 1 and 2

We will conduct 3 exploratory analyses. First, using longitudinal data from baseline, 3, 6, 9 and 12 months, we will perform mixed-effects regression modeling to analyze changes in stigma scores, psychological gender affirmation scores, and GAHT use over time by service delivery model. Mixed-effects regression is most appropriate for clustered longitudinal data as it captures correlations of repeated measures using random effects that describe cluster-specific trends over time [100]. Second, as sample size allows, we will assess HIV outcomes by subgroups. The D-i-D model, used to test hypothesis 1 and hypothesis 2, will be fit separately for subgroups defined by (a) gender identity, (b) sex assigned at birth, (c) individual care facility, and (d) colocated versus stand-alone TG-DSD sites. Third, the association between 12-month HIV outcomes (viral suppression rates and prevention-effective adherence rates) and implementation metrics from aim 1 will be examined graphically for the 4 TG-DSD sites by plotting the HIV outcomes at each site versus mean scores on the AIM, IAM, and FIM, respectively.

The use of D-i-D analyses implicitly assumes that TG-DSD sites are either comparable with SSD sites (other than the DSD approach) or that we can readily adjust for any differences. However, if we find that groups differ on important characteristics, we will use inverse probability of treatment weighting to adjust for the covariates in the effect estimation to mitigate potential bias.

### Data Analysis for Aim 3

We will use the data from the microcosting exercise as well as existing data on SSD costs to estimate the incremental cost of TG-DSD over SSD from the health care system perspective. Integrating data collected for aim 2, we will then calculate the cost per successful outcome (defined as viral suppression for those participants on ART or prevention-effective adherence for the participants who are HIV negative at month 12) and the cost of producing an additional client with a successful outcome. Finally, using any available size estimates for the overall transgender population in South Africa [5], we will then calculate the total budget required for a national rollout of TG-DSD in South Africa, incremental to the country’s HIV budget on which we will have real-time data.

### Statistical Power

Power analyses and sample size calculations were based on the primary outcome of viral suppression (aim 2). No viral load data are available for transgender people in South Africa disaggregated from other key populations. The only South African data that includes transgender women and uses the current nationally recommended threshold for viral suppression includes cisgender men who have sex with men. Nonetheless, the study found a viral suppression rate of 47% [101]. One study of transgender people receiving TG-DSD in South Africa found that those on GAHT were 3 times more likely to be virally suppressed compared to transgender people who are not on GAHT [49]. This suggests an effect size of at least 20%. We consider an effect size as low as 15% to be clinically significant.

On the basis of the aforementioned data and clinical experience, we anticipate that current viral suppression rates will be approximately 65% at baseline, rising to 85% at 12 months among TG-DSD participants, and approximately 61% at baseline, rising to 66% at 12 months among SSD participants. This 20% difference from baseline to 12 months for TG-DSD participants and 5% difference from baseline to 12 months for SSD participants represents a difference in the absolute change in viral suppression rates of 15%. Using *r*=1, α=.05, power of 1− β=.80, and p_1_=viral suppression in the SSD and p_2_=viral suppression in the TG-DSD group and difference of p_2_ – p_1_=.15, the minimum sample per group will be 172 for both TG-DSD and SSD, for a total minimum sample size of 344. Adjusting for loss to follow-up of approximately 12%, a sample of ≥200 in each group, for a total of ≥400 transgender people on ART, provides adequate statistical power for planned analyses.

Loss to follow-up is a challenge for any longitudinal study. We will make every effort to minimize loss to follow-up by collecting multiple forms of contact, reaching out monthly to update contacts, collecting data in the field as well as the study site, and providing remuneration for study participation. We are enrolling a larger sample size than required by the power calculations to allow for potential loss to follow-up without loss of statistical power.

## Results

The Jabula Uzibone study enrollment began in January 2024. As of July 31, 2024, a total of 593 transgender participants have been enrolled: 348 are living with HIV and 245 are HIV negative. We anticipate baseline enrollment will be complete by August 31, 2024, and the final study visit will take place no later than August 2025.

## Discussion

### Expected Findings

We anticipate that the Jabula Uzibone study will demonstrate that TG-DSD is associated with higher rates of viral suppression and prevention-effective adherence compared with SSD. Furthermore, we expect qualitative and quantitative results to indicate that these associations are mediated by gender affirmation and stigma reduction. Importantly, we expect to find that broader implementation of key aspects of TG-DSD models, including cost, are feasible, acceptable, and appropriate.

Prior studies of DSD models support their effectiveness for improving retention in care and viral suppression for people living with HIV in sub-Saharan Africa [102]. However, the largest systematic review assessing DSD implementation barriers and facilitators excluded key populations [103]. This study will fill the critical gap in data on DSD for transgender people, a key population, as well as provide the first data on use of DSD for HIV prevention services.

### Strengths and Limitations

This study is limited by its nonrandomized, observational nature. As TG-DSD is already being offered at Wits RHI sites in South Africa, sites cannot be randomized. While randomized controlled trials (RCTs) are considered the highest standard of scientific evidence, an RCT is not feasible in this situation. If an RCT were possible, it would run the risk of producing findings that cannot be replicated in less controlled settings. Jabula Uzibone will generate rich data on implementation strategies and intervention effectiveness in real-world conditions.

Assessment of prevention-effective adherence among participants who are HIV negative and not prescribed PrEP relies on self-reported sexual behaviors. Recall and social desirability bias may affect the accuracy of self-report. Despite this limitation, our choice to measure overall prevention-effective adherence (rather than limiting measures to tenofovir levels among participants on PrEP) allows for broader applicability of findings to the real lives of transgender people whose sexual behavior and indication for PrEP shift over time [23].

### Conclusions

In addition to dissemination in scientific journals and relevance conferences, results from this study will be shared directly with both transgender community organizations and the National Department of Health in South Africa. Jabula Uzibone will provide actionable data to inform HIV policies and health facility practices in South Africa and generate the first evidence for implementation of TG-DSD in sub-Saharan Africa. Study findings also may inform how other countries could use TG-DSD strategies to increase engagement of transgender people in care and advance global progress toward HIV elimination goals.

## Appendix

Multimedia Appendix 1 Peer review report from the National Institutes of Health.

## Abbreviations

AIM: Acceptability of Intervention Measure
ART: antiretroviral therapy
D-i-D: difference-in-difference
DSD: differentiated service delivery
FIM: feasibility of intervention measure
GAHT: gender-affirming hormone therapy
IAM: Intervention Appropriateness Measure
IDI: in-depth interview
KII: key informant interview
PrEP: pre-exposure prophylaxis
RCT: randomized controlled trial
RE-AIM: Reach, Effectiveness, Adoption, Implementation, and Maintenance
REDCap: Research Electronic Data Capture
SSD: standard service delivery
TG-DSD: transgender-specific differentiated service delivery
UNAIDS: Joint United Nations Programme on HIV and AIDS
WHO: World Health Organization

## Data Availability

The data sets generated during this study will be available from the senior author upon reasonable request 1 year after the completion of data collection.
