# Supplementary material for: Transgender-Specific Differentiated HIV Service Delivery Models in the South African Public Primary Health Care System (Jabula Uzibone): Protocol for an Implementation Study
Source: JMIR Res Protoc. 2024 Sep 13;13:e64373. doi: 10.2196/64373 (PMC11437231; doi:10.2196/64373)
Supplement: Multimedia Appendix 1 [file resprot_v13i1e64373_app1.pdf]

1R01MH130277-01 POTEAT, TONIA

RESUME AND SUMMARY OF DISCUSSION: These applicants seek to assess the impact and effectiveness of implementing a transgender-specific differentiated service delivery as well as the associated costs on ART adherence and viral suppression for transgender persons (TGP) with HIV and PrEP adherence for TGP without HIV. The study which takes advantage of the implementation of demonstration projects in transgender differentiated services in South Africa will be guided by the Gender Affirmation and the RE-AIM frameworks to assess the feasibility and acceptability of specific implementation strategies. The research is significant in that not only are TGP disproportionately represented among persons infected with HIV their engagement in care is suboptimal, and those not infected have very low uptake of PrEP services. The results of this study could inform stakeholders in public health about characteristics of Transgender differentiated services that are most effective in promoting PrEP or sustaining viral suppression. The applicants are very well positioned to conduct this project successfully; they have the necessary training, experience, and complementary skills. The MPI plan is very well justified; the roles of the PIs are well defined and protocols are in place for communication and conflict resolution. The application is theoretically well grounded and the design of the research as well as the analytic plans are very robust. Some minor weaknesses such as the lack of information as to how they will include participants across the 5 sites, the lack of detail regarding facilities and resources for the foreign sites, and the low effort level of the MPIs on the project barely reduced the committee's very high enthusiasm for a project, it feels will have very high impact on identifying effective strategies in the deployment of transgender-specific differentiated services.

DESCRIPTION (provided by applicant): Transgender-specific Differentiated HIV Care Models: An Implementation Science Study Project Summary/Abstract Despite a disproportionate burden of HIV, transgender people are less likely to achieve viral suppression than the general population and have low engagement in PrEP services. Intervenable barriers to their engagement in HIV services include facility-based stigma and lack of access to gender affirming care, including hormone therapy. Transgender-specific differentiated service delivery models have recently been implemented as demonstration projects in South Africa, providing a unique opportunity to assess feasible and acceptable implementation strategies as well as analyze the effectiveness and cost of integrating gender affirming hormone therapy and stigma-reduction strategies into HIV care for transgender people. This observational, multi-site, mixed methods prospective implementation study will be guided by the Gender Affirmation Framework and the RE-AIM (Reach, Effectiveness, Adoption, Implementation, Maintenance) implementation science evaluation framework to meet the following aims: (1) assess barriers, facilitators, acceptability, and feasibility of transgender-specific differentiated service delivery using site observation checklists, key informant interviews with facility staff, and longitudinal in-depth interviews with transgender clients; (2) evaluate the effect of transgender-specific differentiated service delivery on viral suppression and PrEP adherence - testing stigma and gender affirmation as mediators, using a longitudinal cohort of TGP clients that compares transgender people enrolled at transgender-specific differentiated service delivery sites with transgender people enrolled in standard service delivery sites (200/arm on ART and 100/arm on PrEP for a total N = 600); and (3) estimate the cost associated with transgender-specific differentiated service delivery versus standard service delivery sites using a micro-costing approach to estimate the cost per service user served and per service user successfully treated at transgender-specific differentiated service delivery sites relative to standard service delivery sites, as well as the budget needed for successful South Africa-wide implementation.

PUBLIC HEALTH RELEVANCE: Transgender-specific Differentiated HIV Care Models: An Implementation Science Study PROJECT NARRATIVE Despite a disproportionate burden of HIV, transgender people are less likely to achieve viral suppression than the general population and have

low engagement in PrEP services. Transgender-specific differentiated service delivery models have the potential to overcome barriers to HIV service engagement. This study will advance the science on effective HIV care and prevention strategies for transgender people by assessing the implementation, effectiveness, and cost of transgender-specific differentiated service delivery.

CRITIQUE 1

Significance: 2
Investigator(s): 2
Innovation: 1
Approach: 2
Environment: 2

Overall Impact: This observational, multi-site, mixed method prospective implementation R01 submission proposes to assess barriers, facilitators, acceptability, and feasibility of transgender-specific differentiated service delivery in sub-Saharan Africa (SSA). The study will examine the effect of transgender-specific differentiated service delivery on viral suppression and PrEP adherence, and estimate the cost associated with transgender-specific differentiated service delivery vs. standard service delivery. The ultimate goal of the research is to have effective HIV care and prevention strategies for transgender people that are also cost effective. Scaling up transgender care is much needed, and transgender-specific differentiated service delivery, which would be inclusive of hormone therapy is an innovative model of care. The research team (lead by strong MPIs) has the expertise and research experience to implement the study and the environment is strong with the necessary resources. The study aims are clear and appropriate, as are the analyses. A minor weakness in the approach is limited information regarding the recruitment of the target population. The impact is high given the current rates of service utilization for HIV prevention and care for transgender populations.

1. Significance:

Strengths

- Transgender individuals bear a high burden of HIV infection, are less likely to achieve viral suppression, and have low engagement in prevention services (e.g., PrEP).
- Facility-based stigma and the lack of gender affirming care (including hormone therapy) are significant barriers to comprehensive care for this population.
- Transgender-specific differentiated service delivery models are needed to increase uptake of HIV care and prevention services for this population.

Weaknesses

- Research with transgender populations has increased, including the focus on service provision.

2. Investigator(s):

Strengths

- The MPIs (Drs. Poteat and Pettifor) have the expertise and experience to lead this study.
- Dr. Poteat’s (Contact PI: UNC) research is central to the proposed study (HIV inequities among transgender adults), nationally and internationally. She is a key member of the UNC CFAR and leads the Transgender Health Working Group.

- Dr. Pettifor (UNC) has research experience along the HIV care continuum with adolescents and young adults globally, while Dr. Nash (Wits Health) provides treatment and prevention service for vulnerable populations, including transgender individuals.
- The Co-I (Health Economist) and Consultant (Biostatistician) complete the research team.
- The MPI plan is detailed and appropriate.

Weaknesses

- There is some overlap in expertise between Drs. Poteat and Pettifor.

3. Innovation:

Strengths

- The focus on inclusive transgender populations (TM, TW, and nonbinary) in SSA.
- The proposal of comprehensive transgender-specific differentiated service delivery in SSA.

Weaknesses

- None noted.

4. Approach:

Strengths

- The study is guided by the Gender Affirmation Framework and the RE-AIM frameworks.
- Preliminary studies with transgender populations inclusive of mixed methods, PrEP, and the assessment of gender affirming HIV prevention and care services.
- Multi-site, multi-method study, with appropriate study aims and analyses.
- Transgender individuals will be hired as community health workers and peer navigators.
- Comparison of transgender-specific care to standard of care.
- Evaluation of cost associated with transgender-specific differentiated service delivery.
- Sex as a biological variable is addressed.

Weaknesses

- Recruitment of 600 transgender individuals may be challenging, even with 5 sites given facility-based stigma and the low HIV care and prevention service utilization for the population compared to the general population (as well as the previous recruitment of the population for studies). More information on recruitment methods would have been helpful.

5. Environment:

Strengths

- The University of North Carolina Chapel Hill is excellent to support successful implementation of the study.

Weaknesses

- Information on Facilities and Resources was missing for Wits Health Consortium (and/or study sites). However, Letters of Support were included.

Study Timeline:

Strengths

- The timeline is detailed and appropriate with regard to start-up, recruitment, enrollment, follow-up, and dissemination.

Weaknesses

- None noted.

Protections for Human Subjects:

Acceptable Risks and/or Adequate Protections

- Human Subjects Protections are addressed.

Data and Safety Monitoring Plan (Applicable for Clinical Trials Only):

Acceptable

- The plan addresses all safety concerns.

Inclusion Plans:

- Sex/Gender: Distribution justified scientifically
- Race/Ethnicity: Distribution justified scientifically
- For NIH-Defined Phase III trials, Plans for valid design and analysis: Not applicable
- Inclusion/Exclusion Based on Age: Distribution justified scientifically
- Transgender individuals age 18 and older will be recruited.

Vertebrate Animals:

Not Applicable (No Vertebrate Animals)

Biohazards:

Not Applicable (No Biohazards)

Applications from Foreign Organizations:

Justified

- The study focuses on transgender-specific differentiated service delivery in SSA.

Resource Sharing Plans:

Acceptable

- There is a detailed plan for sharing resources.

CRITIQUE 2

Significance: 1
Investigator(s): 1
Innovation: 1
Approach: 2
Environment: 3

Overall Impact: This R01 application from a seasoned investigative team working in an outstanding environment seeks to evaluate the implementation of differentiated service delivery (DSD) for transgender people (TGP) in South Africa and assess the impact of TG-TSD on viral suppression and PrEP adherence, testing stigma and gender affirmation as mediators. The proposed study will also estimate the cost associated with providing TG-DSD vs. standard service delivery. The significance and innovation of the proposed research are high, as the scientific knowledge generated would substantially advance the field, given the dearth of information on this topic. The implementation science approach is strong, using appropriate frameworks, a difference-in-difference design, and longitudinal qualitative interviews.

1. Significance:

Strengths

- Transgender people (TGP) are a key population for HIV prevention and treatment efforts.
- Profound inequities exist with regard to TGP and HIV outcomes in South Africa, where little research has been conducted with this key population.
- Addressing facility stigma towards TGP and providing gender-affirming hormone treatment represent promising approaches to improve care engagement.
- The proposed study seeks to conduct research on the implementation, effectiveness, and cost of providing these services to TGP via real world demonstration projects.

Weaknesses

- None noted by reviewer.

2. Investigator(s):

Strengths

- Dr Poteat has pioneered HIV-related research with transgender women in South Africa.
- Dr Pettifor is an accomplished implementation science expert with a robust research portfolio in South Africa, including a D43 training grant.
- South African collaborators bring expertise in HIV medicine and GAHT, cost analysis, and statistics.

Weaknesses

- None.

3. Innovation:

Strengths

- Assessing the impact of the provision of GAHT for TGP on HIV prevention and treatment outcomes in South Africa is innovative.
- The research will seek to disaggregate data by transgender women, transgender men, and nonbinary gender.

Weaknesses

- None noted by reviewer.

4. Approach:

Strengths

- Preliminary data from local context strongly suggests use of GAHT is associated with greater odds of viral suppression.
- Use of Gender Affirmation Framework and RE-AIM.
- Difference in difference approach to comparing TG-DSD sites to SSD sites.
- Ability for patient participants to have study visits at the facility or on mobile units in the community is a strength.
- Detailed qualitative analysis plan that will draw on both template analysis and standard coding and accounts for the longitudinal nature of the data.
- Limitations section acknowledges potential use of IPTW and loss to follow up, as well as switching from SSD to TG-DSD site.

Weaknesses

- The strategy of community-based recruitment via peers is a little confusing – as the goal of the study is to assess the receipt of service, it is not clear why facility-based recruitment isn’t the primary mode of recruitment. It is also possible that community recruitment could identify people not in care - will these individuals be directed to SSD or TG-DSD sites?
- In addition, it is not clear whether individuals will be recruited as they are establishing care at these sites. Will time in care at site be assessed in the analysis?
- D-i-D analysis plan mentions regressing VS on 6 month time point but viral load is not measured at 6 months.

5. Environment:

Strengths

- UNC has resources to support global health research.
- Appropriate letters of support from local health authorities.

Weaknesses

- No facilities and resources section for the Wits Health Consortium or any of the South African sites where study work will be conducted.

Study Timeline:

Strengths

- None noted by reviewer.

Weaknesses

- None noted by reviewer.

Protections for Human Subjects:

Acceptable Risks and/or Adequate Protections

- Acceptable.

Data and Safety Monitoring Plan (Applicable for Clinical Trials Only):

Acceptable

Inclusion Plans:

- Sex/Gender: Distribution justified scientifically
- Race/Ethnicity: Distribution justified scientifically
- For NIH-Defined Phase III trials, Plans for valid design and analysis: Not applicable
- Inclusion/Exclusion Based on Age: Distribution justified scientifically

Vertebrate Animals:

Not Applicable (No Vertebrate Animals)

Biohazards:

Unacceptable

- Need description of collection/safe handling of DBS.

Applications from Foreign Organizations:

Justified

Resource Sharing Plans:

Acceptable

- South African qualitative lead has 0% effort in Years 2 and 3 and no qualitative interviewer time is budgeted in Year 2, when the qualitative interviews are slated to begin. Similarly, the project administrator with day-to-day oversight of the study has 0% effort in Years 3 and 4.
- Some of the South African budget justification may be from another grant application, as it references examination beds, anal screening speculums and pap smear speculums.

CRITIQUE 3

Significance: 1
Investigator(s): 1
Innovation: 2
Approach: 1
Environment: 1

Overall Impact: This new submission R01 seeks to evaluate the implementation and effectiveness of strategies that overcome barriers to HIV service engagement for transgender persons (TGP). The problem is of high significance given that TGP are key populations in regard to HIV elimination goals and that there is a dearth of evidenced based interventions available. The study will be performed in South Africa, which is justified given the significant burden of disease in that country and the first region to implement DSD for TGP. The study design is observational, multi-site mixed methods and prospective, and outcomes include implementation metrics (including costs) and effectiveness of the TG- DSD compared to TG-SSD. The PI and research team are excellent and have key expertise / positions related to transgender health and research, South African HIV policy and implementation science. The proposal is highly innovative given the sole focus on TGP with attention to TM, the inclusion of the full continuum of HIV care including PrEP, and the incorporation of GAHT. The approach well leverages the natural experiment of TG-DSD roll out in South Africa, and potential limits of observational data (biases etc.) will be explored through thorough understanding of implementation issues at the facility and individual level. The team has prior experience with successful recruitment in the population, collaboration with the existing health system and a number of the study instruments which reflects on likely feasibility. The use of Gender Affirming and RE-AIM frameworks is appropriate and the analytical plan is well thought-out. Overall, this is an exceptional application from a highly experienced team that has the potential for high impact in identifying models and their related implementation issues for providing differentiated care for a vulnerable and understudied key population.

1. Significance:

Strengths

- Limited but compelling data exist on GAHT in DSD for TGP – what that do exist are single site, case series and cross sectional and have not been performed in the Sub-Saharan setting. The study seeks to evaluate the effectiveness and implementation of DSD for TGP in a more robust fashion in a high prevalence region.
- Robust evidence for non- KP DSD demonstrates effectiveness in improving viral suppression and retention in care compared to SSD, as well as reduced costs.

Weaknesses

- None.

2. Investigator(s):

Strengths

- The PI is an excellent senior researcher with extensive expertise in community engaged, social research on HIV in TGP globally.
- The skillset of the investigative team is complementary and includes implementation research, expertise and gender-affirming clinical care in the local setting, cost analysis and biostatistics.

Weaknesses

- None.

3. Innovation:

Strengths

- The proposal examines strategies for TGP as the sole focus of study. Additionally, TM are understudied in HIV research, and the inclusion of this population is unique.
- The proposal will examine the full continuum of care, inclusive of PrEP.
- The study will be the first to incorporate GAHT in Sub-Saharan Africa and will be using a robust, multi-site comparative design.

Weaknesses

- None.

4. Approach:

Strengths

- Preliminary studies led by the PI on TGP in South Africa provide motivation for the proposal, including low rates of PrEP awareness, GAHT uptake, and high rates of healthcare related stigma. In those TGP with HIV who did access to GAHT, the odds of viral suppression were 2.4 more than those who did not access GAHT, suggesting the positive impact of gender affirming care on HIV care engagement.
- Prior reports of number of TGP receiving care at study sites (>2000) supports feasibility of study sample size.
- The study design, including the frameworks (RE-AIM) are well chosen to leverage the timing and opportunity that DSD will be implemented by the ministry of health.
- The Gender Affirmation Framework is suited to inform the hypothesis that TG-DSD will be more effective in patient engagement and HIV outcomes based on the theory that this strategy can reduce anti-transgender stigma that drives social oppression / psychological distress and results in non-engagement.
- Tools and procedures for data collection including survey instruments and social media based recruitment have been successfully used in a prior study (TMAPP) which supports feasibility.
- The analytical plan, including qualitative, quantitative, triangulation / data integration of the aforementioned using joint display, mediation analysis and power calculations are well described, strengthening the interpretability of findings.

- Justification for non RCT study is appropriate, given the pragmatic / natural experiment conditions requiring an observational design.
- Sex as a biological variable is well considered in this study, as are potential limitations and methods of mitigation.

Weaknesses

- It is unclear which sites provide care through mobile units, which may address some structural barriers to engagement and bias results. This may be addressed in the analysis, dependent on sample size.

5. Environment:

Strengths

- UNC is an excellent environment for this study, and includes robust research support through the CPC, OSR, and the CFAR.
- The South African partners have a history of prior, successful collaboration.

Weaknesses

- None.

Study Timeline:

Strengths

- The study timeline appropriately considers start up activities, recruitment and retention and is reasonable.

Weaknesses

- None.

Protections for Human Subjects:

Acceptable Risks and/or Adequate Protections

- TGP / clinic staff protections / risk mitigation well laid out.

Data and Safety Monitoring Plan (Applicable for Clinical Trials Only):

Acceptable

- No concerns.

Inclusion Plans:

- Sex/Gender: Distribution justified scientifically
- Race/Ethnicity: Distribution justified scientifically
- For NIH-Defined Phase III trials, Plans for valid design and analysis: Not applicable
- Inclusion/Exclusion Based on Age: Distribution justified scientifically

- Sex/gender (TGP) is justified given focus of study and research gap. Race/ethnicity is justified based on local demographics. Age is justified given particular needs of TG adolescents being beyond the scope of the clinic sites.

Vertebrate Animals:
Not Applicable (No Vertebrate Animals)

Biohazards:
Not Applicable (No Biohazards)

Applications from Foreign Organizations:
Justified

- South African setting is justified by burden of disease and TG-DSD clinical implementation.

Resource Sharing Plans:
Acceptable

- Data will be shared pending appropriate DUAs.

Authentication of Key Biological and/or Chemical Resources:
Not Applicable (No Relevant Resources)

THE FOLLOWING SECTIONS WERE PREPARED BY THE SCIENTIFIC REVIEW OFFICER TO SUMMARIZE THE OUTCOME OF DISCUSSIONS OF THE REVIEW COMMITTEE, OR REVIEWERS' WRITTEN CRITIQUES, ON THE FOLLOWING ISSUES:

PROTECTION OF HUMAN SUBJECTS: ACCEPTABLE

INCLUSION OF WOMEN PLAN: ACCEPTABLE

INCLUSION OF MINORITIES PLAN: ACCEPTABLE

INCLUSION ACROSS THE LIFESPAN: ACCEPTABLE
